# Gatekeepers of ovarian fate: the molecular landscape of diminished ovarian reserve

**DOI:** 10.3389/fendo.2026.1926422

**Published:** 2026-10-26

**Authors:** Li Tang, Long Zhou, Guanghui Cui, Jiacheng Zhang, Hangqi Hu, Ruifan Lin, Yuxin Jin, Ze Ma, Xichen Chai, Lan Wang, Yutian Zhu, Xiyan Xin, Yang Ye, Dong Li

**Affiliations:** 1 State Key Laboratory of Female Fertility Promotion, Department of Traditional Chinese Medicine, Peking University Third Hospital, Beijing, China; 2 Department of Traditional Chinese Medicine, Peking University People’s Hospital, Beijing, China; 3 Department of Integrated Traditional Chinese and Western Medicine, Peking University First Hospital, Beijing, China

**Keywords:** cell death, diminished ovarian reserve, follicle development, genetics and epigenetics, molecular mechanisms, ovarian aging, oxidative stress

## Abstract

Diminished ovarian reserve (DOR) is a major reproductive disorder characterised by accelerated follicular depletion and impaired ovarian responsiveness, yet its molecular basis remains incompletely understood. This review integrates current evidence across the major biological processes that determine ovarian reserve and follicular competence. Primordial follicle activation governed by PI3K–AKT–FOXO3/PTEN–mTOR, Hippo–YAP, KIT/KITL and AMH signaling represents an upstream determinant of follicle-pool consumption, while bidirectional oocyte–granulosa cell communication and the stromal microenvironment sustain follicular development. Genomic instability, defective DNA repair, chromatin remodeling and epigenetic dysregulation further compromise cellular integrity and reproductive longevity. Mitochondrial bioenergetic failure, mtDNA instability, altered mitochondrial dynamics and oxidative stress form a central mechanistic hub linking metabolic dysfunction to follicular damage. Emerging evidence additionally highlights the autophagy–mitophagy–lysosome system, including stress-granule clearance and lysosomal proteostasis, as a context-dependent quality-control network rather than an intrinsically lethal pathway. Downstream cell-fate responses, particularly granulosa-cell apoptosis, cellular senescence and ferroptosis, together with immune–inflammatory and endocrine disturbances, further promote follicular attrition. Importantly, the strength of evidence varies substantially across mechanisms, with direct human DOR data available for only a subset of pathways and many proposed mechanisms remaining dependent on animal or cellular models. Clinically, AMH and antral follicle count remain the principal reserve markers, whereas molecular biomarkers and targeted interventions are largely investigational. By organizing these mechanisms into an integrated framework, this review identifies key evidence gaps and priorities for mechanistically informed biomarker development and therapeutic translation in DOR.

## 1 Introduction

Diminished ovarian reserve (DOR) is best regarded as a clinical phenotype of reduced quantitative ovarian reserve relative to chronological age, typically reflected by lower anti-Müllerian hormone (AMH) concentrations, a reduced antral follicle count (AFC), and/or a lower oocyte yield after ovarian stimulation. Importantly, ovarian-reserve tests estimate follicle/oocyte quantity rather than oocyte quality: AMH and AFC predict ovarian response more reliably than reproductive potential independently of age, and neither should be interpreted as a stand-alone fertility test (1). This distinction is particularly important because DOR is frequently discussed alongside poor ovarian response (POR), premature ovarian insufficiency (POI), the older term premature ovarian failure (POF), and physiological ovarian ageing, although these phenotypes are not interchangeable. POR is a stimulation-response construct defined by the Bologna criteria and further stratified by the POSEIDON framework (2, 3). POI, by contrast, denotes loss of ovarian activity before 40 years of age; the updated international evidence-based guideline defines it by disordered menstrual cycles for at least four months together with an FSH concentration >25 IU/L, with repeat testing after 4–6 weeks recommended only when diagnostic uncertainty remains (4). Physiological ovarian ageing describes the expected age-associated decline in reserve and reproductive competence. These distinctions are summarised in **Table 1** and are maintained throughout this review so that mechanistic evidence is not extrapolated across phenotypes without qualification.

**Table 1:** Diagnostic comparison of DOR, POR, POI/POF and physiological ovarian ageing.

Feature	DOR	POR (Bologna/POSEIDON)	POI/POF	Physiological ovarian ageing
What it describes	Reduced quantitative ovarian reserve relative to age; oocyte quality is not part of the definition	Reduced oocyte yield in a specific IVF stimulation cycle	Loss of ovarian activity before age 40	Age-concordant decline in reserve, occurs in essentially all women
Typical age	Any age; more common after 35	Any age meeting criteria	By definition < 40 years	Progressive from the mid-30s onward
Core diagnostic marker(s)	AMH/AFC and, in stimulated cycles, oocyte yield; markers of quantity and poor independent predictors of reproductive potential	Bologna: ≥2 of (age ≥40 or POR risk factor; ≤3 oocytes in a prior conventional cycle; AFC <5–7 or AMH <0.5–1.1 ng/mL). POSEIDON: 4 groups stratified by age + reserve + prior response	Disordered menstrual cycles (amenorrhoea or irregular cycles) ≥4 months plus FSH >25 IU/L; repeat FSH after 4–6 weeks only if diagnostic uncertainty remains	AMH/AFC decline tracking chronological age; no fixed cut-off
Menstrual cycles	Usually regular	Not part of the definition (a stimulated-cycle response measure)	Irregular or absent	Regular until perimenopause
Spontaneous pregnancy possible?	Often, though chance is reduced	Describes a cycle response, not fecundity per se	Yes in a subset, via intermittent follicular activity	Yes, with declining probability
Typical ART/IVF outcome	Lower oocyte yield than age-matched peers; pregnancy and live-birth prognosis remains strongly influenced by age and embryo/oocyte competence (ref. 1)	Fewer oocytes retrieved in the index cycle; cumulative delivery rate roughly 50% lower than normal responders across POSEIDON groups (ref. 5)	Natural conception remains possible in a minority (~5–10%); donor oocytes are the most effective option when it does not occur (ref. 6)	Outcome tracks chronological age; no diagnosis-specific prognosis beyond age

The biological basis of ovarian reserve extends beyond the size of the remaining follicle pool. Its trajectory reflects the rate at which primordial follicles leave quiescence, the ability of activated follicles to maintain oocyte–granulosa cell communication and metabolic support, and the capacity of ovarian cells to preserve genomic, epigenetic, mitochondrial and proteostatic integrity under chronic physiological and environmental stress. Mouse genetic studies establishing FOXO3 and PTEN as key restraints on primordial-follicle activation, together with work linking granulosa-cell mTOR-dependent metabolism to follicle recruitment, illustrate how upstream activation control can determine the pace of reserve consumption (5–8). Downstream, DNA damage and repair failure, chromatin and non-coding-RNA dysregulation, mitochondrial dysfunction and oxidative stress, autophagy–lysosome dysfunction, senescence, apoptosis, ferroptosis, immune-inflammatory signalling and systemic endocrine inputs influence whether a recruited follicle remains competent or undergoes attrition. These processes are therefore better viewed as an interconnected hierarchy of ‘gatekeepers’ than as independent pathway lists.

This article was developed as a structured narrative review aimed at mechanistic integration rather than pooled effect estimation. PubMed/MEDLINE and the Web of Science Core Collection were searched from database inception to 15 September 2026. Search concepts combined terms for diminished ovarian reserve and ovarian reserve with mechanism-specific terms covering primordial-follicle activation and quiescence; PI3K–AKT–FOXO3/PTEN–mTOR, Hippo and KIT/KITL signalling; oocyte–granulosa communication; DNA damage and repair; genomic instability; chromatin remodelling; DNA and RNA methylation; histone modification; non-coding RNAs; mitochondrial bioenergetics and dynamics; mtDNA; autophagy, mitophagy and lysosomal proteostasis; oxidative stress and antioxidant defence; apoptosis, ferroptosis and cellular senescence; inflammation, cytokines and inflammasomes; extracellular-matrix remodelling, fibrosis and vascular ageing; and endocrine or systemic modulators. Reference lists of included studies and authoritative reviews were screened to identify additional relevant reports. Peer-reviewed human DOR studies were prioritised, followed by closely related human ovarian phenotypes, mammalian causal models and ovarian cell or tissue studies. Non-ovarian or non-mammalian evidence was retained only when it clarified a general mechanism not adequately resolved in ovarian systems and is explicitly labelled as indirect. Duplicate reports, plant studies and cross-organ references without direct relevance to an ovarian claim were excluded. Because the literature spans heterogeneous phenotypes, models and endpoints, no quantitative meta-analysis was attempted.

Within this framework, we organise the molecular landscape of DOR into interconnected gatekeeper domains: primordial follicle activation; oocyte–granulosa cell communication and the follicular microenvironment; genomic stability and DNA repair; chromatin and epigenetic regulation; mitochondrial bioenergetics and redox homeostasis; autophagy–lysosome quality control; cell fate, senescence and regulated cell death; and immune-inflammatory and endocrine/systemic modulation. We then assess how these mechanisms intersect, where direct human DOR evidence is strongest or weakest, and which biomarkers or interventions have progressed beyond mechanistic plausibility toward clinical validation. The goal is to provide a causally organised and evidence-aware framework for interpreting DOR biology and for prioritising the human studies needed to translate molecular insight into more reliable reproductive counselling and treatment. The integrated framework and its evidence hierarchy are summarised in **Figure 1**.

**Figure 1:**
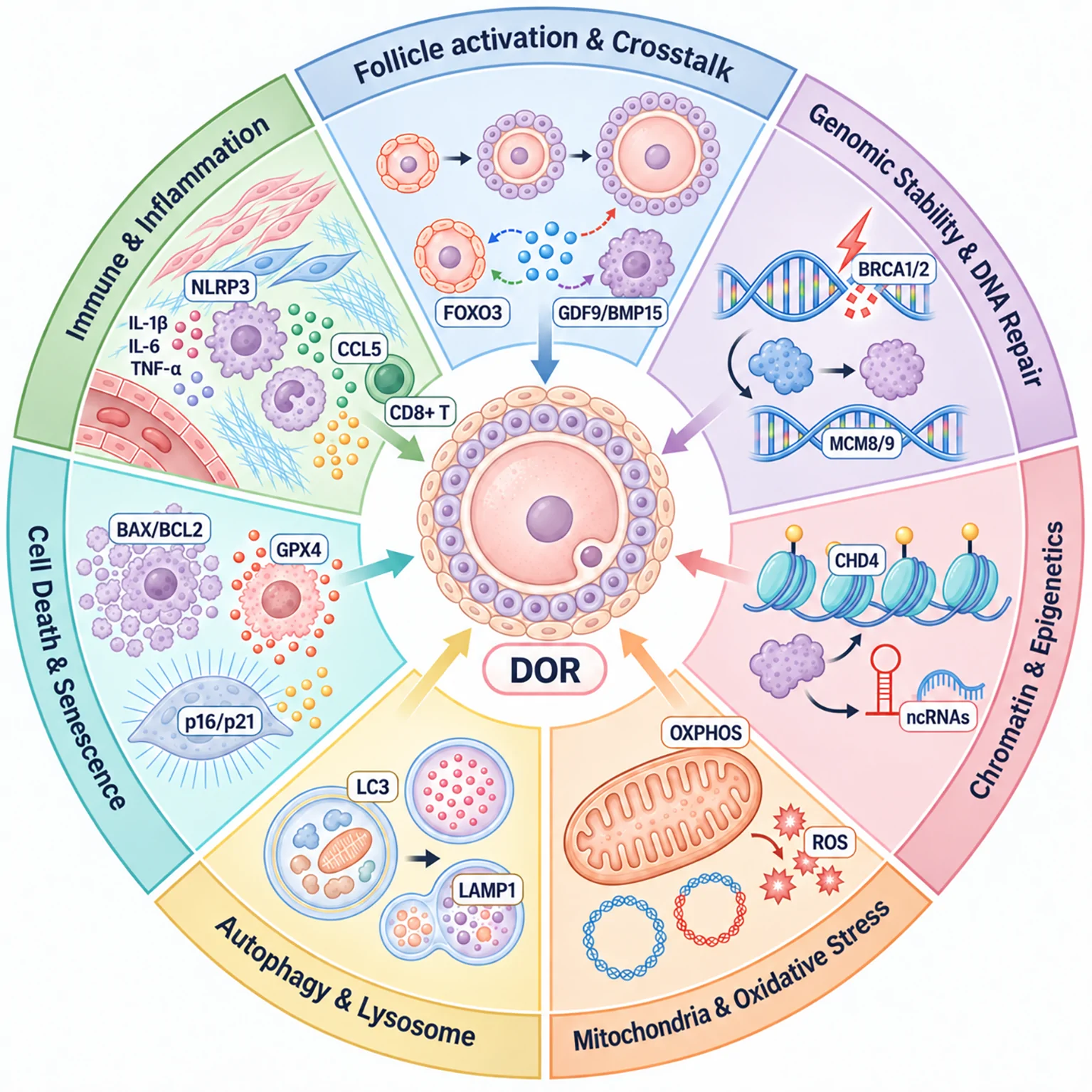
Integrated molecular framework of diminished ovarian reserve. Diminished ovarian reserve (DOR) arises from interconnected alterations in follicle activation and oocyte-granulosa crosstalk, genomic stability and DNA repair, chromatin and epigenetic regulation, mitochondrial and redox homeostasis, the autophagy-lysosome system, cell fate and regulated cell death, and immune-inflammatory signalling. Together, these processes influence follicular maintenance, cellular quality control and progressive depletion of the ovarian reserve.

## 2 Molecular mechanisms of diminished ovarian reserve

### 2.1 Primordial follicle quiescence and activation

The finite, non-renewing primordial follicle pool established before or shortly after birth is the physical substrate of “ovarian reserve”; how fast that pool is drawn down is set primarily by the balance between quiescence-maintaining and activation-promoting signalling inside the oocyte and its surrounding pre-granulosa cells, not simply by chronological time. This gatekeeping step has received comparatively little attention relative to downstream cell-death pathways in much of the DOR mechanistic literature, despite arguably acting further upstream in the causal chain that ultimately determines reserve.

#### 2.1.1 The PI3K-AKT-FOXO3 and PTEN-mTOR axis

Within the oocyte, the PI3K–AKT–FOXO3 axis is the best-characterised brake on activation. FOXO3 is a forkhead transcription factor that, while dephosphorylated and nuclear, keeps primordial follicles dormant; oocyte-specific deletion of *Foxo3* in mice causes global, synchronous activation of the entire primordial pool followed by early depletion and infertility (5, 6). PTEN is the lipid phosphatase that restrains PI3K/AKT signalling upstream of FOXO3; oocyte-specific *Pten* deletion produces the same phenotype of pool-wide premature activation (7). Downstream, mTORC1 activity in KIT-ligand (KITL)-producing pre-granulosa cells couples to granulosa-cell glycolysis to drive the primordial-to-primary transition, linking metabolic state directly to activation rate (8), with confirmation that the same glycolytic increase occurs during human primordial-to-primary transition. PPARγ modulators acting through the PTEN/AKT/FOXO3a axis have been shown to accelerate or retard neonatal mouse follicle activation *in vitro*, providing pharmacological proof of concept for this pathway (9).

#### 2.1.2 The Hippo-YAP pathway

The Hippo–YAP pathway provides a second, largely independent brake that operates at the level of ovarian tissue mechanics rather than single-oocyte signalling. Mechanical disruption (fragmentation) of ovarian cortex increases actin polymerisation, inactivates Hippo signalling, and drives nuclear translocation of the effector YAP, which in turn up-regulates growth factors (CCN family) that promote follicle growth; in a landmark translational study, ovarian fragmentation combined with a small-molecule AKT stimulator was used to activate residual follicles in women with primary ovarian insufficiency, with oocyte retrieval, fertilisation and a live birth reported in one patient (10) — one of the few direct human proof-of-concept demonstrations of this pathway, though the cohort was small and uncontrolled.

#### 2.1.3 AMH and KIT/KITL signalling

AMH, secreted by granulosa cells of already-growing (secondary and early antral) follicles, feeds back to *inhibit* recruitment of neighbouring primordial follicles; AMH-null mice show accelerated recruitment, a larger transient pool of growing follicles, and earlier depletion of the primordial reserve (11). This is mechanistically important because it means the single most widely used clinical biomarker of ovarian reserve (AMH) is not a passive marker but an active *regulator* of the process it is used to monitor — a point that is frequently lost when AMH is discussed purely as a diagnostic number. KIT/KITL signalling between the oocyte and pre-granulosa cells is required for both survival and activation of primordial follicles and interacts with the PI3K/AKT and mTORC1 nodes above; a recent comprehensive update on initial and cyclic follicle recruitment integrates these pathways together with Notch (Jagged1) signalling between neighbouring pre-granulosa cells (12).

Overall, the PTEN–AKT–FOXO3 and mTORC1–KITL circuitry governing follicle activation is established almost entirely in mouse genetic models and *in vitro* human ovarian-cortex culture, whereas the Hippo-disruption/AKT-stimulator approach described above remains the field’s clearest direct human proof of concept, albeit from small, uncontrolled case series; whether such *in vitro* activation meaningfully increases live birth in unselected women with DOR, as opposed to POI, has not yet been established.

### 2.2 Oocyte–granulosa cell communication and the follicular microenvironment

Oocytes are not autonomous; follicle survival depends on continuous bidirectional signalling with surrounding granulosa cells, an aspect of ovarian-reserve regulation that is often underemphasised relative to intra-oocyte or intra-granulosa-cell mechanisms considered in isolation.

#### 2.2.1 GDF9, BMP15 and gap-junction communication

Oocyte-secreted growth differentiation factor 9 (GDF9) and bone morphogenetic protein 15 (BMP15) are TGF-β superfamily ligands that instruct granulosa-cell proliferation, differentiation and metabolic support of the oocyte; loss-of-function of either is associated with arrested folliculogenesis in mouse models and, in humans, with variants linked to POI and DOR phenotypes (13). GDF9 and BMP15 have divergent, sometimes opposing, effects on gap-junction communication: BMP15, but not GDF9, down-regulates connexin43 (Cx43) and reduces gap-junction intercellular communication in human granulosa cells via a SMAD-dependent pathway (14). However, this directional result should therefore be interpreted cautiously and not treated as definitive evidence. Connexin37 (oocyte–granulosa) and connexin43 (granulosa–granulosa) gap junctions, together with transzonal projections (TZPs) — thin cytoplasmic extensions from cumulus cells that cross the zona pellucida to contact the oocyte membrane — form the physical conduits for metabolic coupling (pyruvate, amino acids, cAMP) and for oocyte-derived paracrine signals that in turn regulate cumulus-cell metabolism and cholesterol supply (15). Loss of this coupling, whether through connexin deficiency, TZP retraction with follicle maturation, or oocyte-derived signal deficiency, is associated with impaired oocyte developmental competence across species.

#### 2.2.2 Stromal fibrosis, extracellular matrix and vascular ageing

Beyond the individual follicle, the stromal compartment sets the microenvironment in which follicles must survive: ovarian collagen deposition increases and hyaluronan content decreases with age in both mouse and human ovary, increasing tissue stiffness in a manner proposed to physically constrain follicle growth and to alter the mechanotransduction inputs that feed into the Hippo pathway described above (16). Single-cell and spatial transcriptomic atlases of the human ovary have begun to resolve stromal, immune, vascular and follicular compartments simultaneously and identify extracellular-matrix remodelling as a defining feature of the ovarian cortex (17), and a parallel single-cell atlas of the aging mouse ovary offers a cross-species resource for testing whether stromal ageing signatures co-localise with follicle loss (18); ovarian vascular ageing (reduced stromal microvascular density and altered angiogenic signalling) has similarly been linked to reduced oxygen and nutrient delivery to growing follicles. This remains a largely descriptive literature relative to the causal evidence available for follicle-intrinsic pathways.

Taken together, GDF9/BMP15 pathway variants are directly documented in human POI and DOR cohorts, whereas the connexin/gap-junction and transzonal-projection data derive from human granulosa-cell lines and primary cells rather than from intact follicles *in situ*, and the stromal fibrosis, extracellular-matrix, and vascular-ageing evidence — while now including direct human tissue data on stiffness and matrix composition — remains predominantly descriptive rather than causal; whether such stromal changes are a cause of follicle loss or simply fill the space left by follicles already lost remains an open question. The principal follicle-activation and oocyte–granulosa communication pathways are summarised in **Figure 2**.

**Figure 2:**
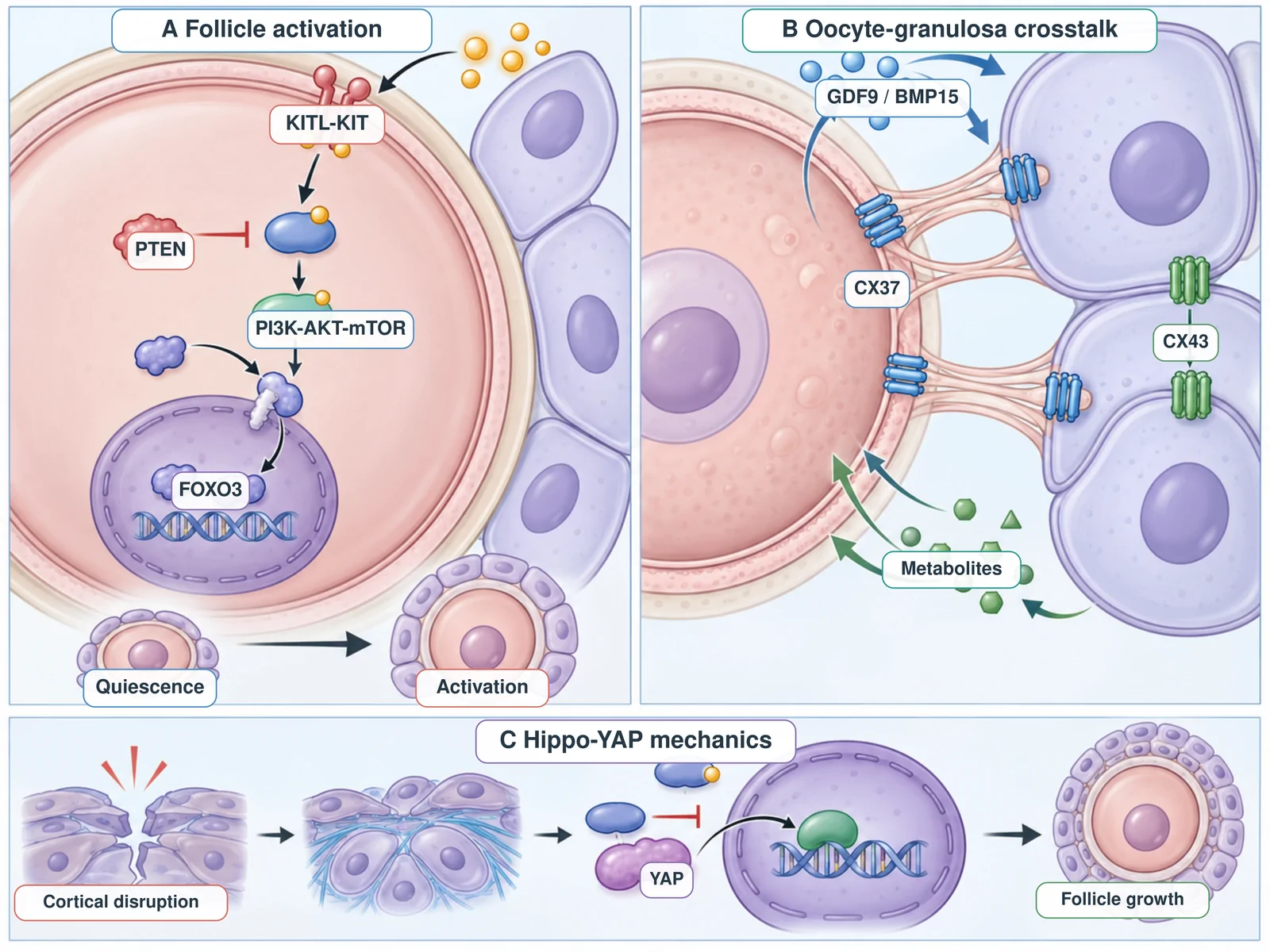
Follicle activation and oocyte-granulosa crosstalk. **(A)** Primordial follicle quiescence and activation are regulated by KITL-KIT signalling and the PTEN-PI3K-AKT-mTOR-FOXO3 axis. **(B)** Oocyte-derived GDF9 and BMP15, connexin-mediated gap junctions and metabolite exchange support bidirectional communication between the oocyte and surrounding granulosa cells. **(C)** Changes in ovarian cortical mechanics can modulate Hippo-YAP signalling and promote follicle growth. These pathways collectively regulate recruitment from the primordial follicle pool and subsequent follicular development.

### 2.3 Genomic stability and DNA repair

#### 2.3.1 DNA damage burden in ageing oocytes and granulosa cells

Human oocytes are formed prenatally, arrest in meiotic prophase I, and may remain dormant for decades before being recruited for growth; this longevity makes DNA damage accumulation and repair capacity central to ovarian reserve. Aged oocytes show increased markers of DNA double-strand breaks (DSBs, e.g. γH2AX foci) together with a reduced capacity for homologous recombination (HR) repair, as demonstrated directly in aged human/porcine oocytes and linked to deteriorating cohesin and DNA-repair-compartment organisation (19, 20). Insufficient recruitment of telomeric DNA-damage-response factors (RNF8, 53BP1) has been linked to telomere-driven chromosomal instability in aged oocytes (21, 22). The kinase CHEK2 and its downstream effectors p53/TAp63 form the “DNA damage checkpoint” that eliminates primordial follicles carrying unrepaired DSBs, illustrated most directly by single-cell/bulk transcriptional profiling of the mouse ovarian DNA-damage response (23).

#### 2.3.2 Key DNA repair pathways

Two canonical pathways repair DSBs: error-free HR, which requires a homologous template and is active in S/G2 and during meiotic recombination (mediated by RAD51 loading onto resected ends, itself catalysed by the MRE11–RAD50–NBS1 complex and coordinated with ATM checkpoint signalling) (24–27), and error-prone non-homologous end joining (NHEJ, Ku70/Ku80–DNA-PKcs–ligase IV), whose balance against HR is set by 53BP1 (pro-NHEJ) versus BRCA1 (pro-HR, particularly in replicated chromatin) (28–30). The relative use of these pathways has been examined in mouse oocytes during meiotic maturation and during embryonic/perinatal ovarian-reserve formation (31, 32). Alternative, more mutagenic end-joining routes (microhomology-mediated end joining, single-strand annealing) are used when resection is extensive, and chromatin context (regulated in part by the oocyte-differentiation transcription factor TAF4b) and RNA: DNA hybrid resolution (XRN2) further shape pathway choice (33–35).

#### 2.3.3 Genetic lesions linked to DOR

Genetic lesions in this pathway map directly onto human DOR/POI. Biallelic BRCA1 frameshift mutations have been found in young women with isolated DOR without other Fanconi-anaemia features, and BRCA1/BRCA2 carriers show accelerated, chemotherapy-exacerbated ovarian reserve loss attributable to impaired oocyte DSB repair (36, 37). BRIP1 mutation increases carboplatin/paclitaxel ovarian toxicity in a mouse carrier model (38). Heterozygous HFM1 (meiotic recombination helicase) variants have been identified in sisters with DOR and recurrent pregnancy loss (39), and a homozygous KASH5 (meiosis-specific LINC-complex) frameshift mutation was found in a consanguineous family with DOR, recurrent miscarriage and non-obstructive azoospermia (40). RNF111, an E3 ligase regulating TGF-β/BMP signalling, shows deleterious monoallelic variants in POI/DOR patients, with haploinsufficient mice recapitulating reduced ovarian reserve (41). Whole-exome sequencing of DOR/POI cohorts continues to expand this polygenic picture, and clinical genetic counselling (including for FMR1 premutations and BRCA variants) is now integral to fertility-preservation decision-making (42–45).

Among these mechanisms, the monogenic causes of impaired DNA repair (BRCA1/2, HFM1, KASH5, RNF111) are directly documented in human DOR and POI cohorts and are among the best-supported findings in this field, whereas cohesin loss and telomeric DNA-damage-response deficits have so far been demonstrated mainly in aged human or mouse oocytes generally rather than in age-matched comparisons of DOR against normal reserve. The relative contribution of stochastic double-strand-break accumulation versus genetically determined repair capacity to an individual woman’s reserve trajectory therefore remains unresolved, as does most of the detailed pathway-choice biochemistry, which has been characterised largely outside the oocyte.

### 2.4 Chromatin remodeling and epigenetic regulation

Chromatin state, DNA methylation, histone modification and non-coding RNA (ncRNA) networks together govern which genes an oocyte or granulosa cell can express at each stage, and dysregulation of any layer has been linked to follicle loss.

#### 2.4.1 Chromatin dynamics and remodellers

Mouse germinal-vesicle oocytes exist in two chromatin configurations — transcriptionally permissive “non-surrounded nucleolus” (NSN) and transcriptionally silent “surrounded nucleolus” (SN) — and the NSN-to-SN transition, which stores maternal mRNA for use after fertilisation, is a prerequisite for developmental competence (46, 47). Histone H3K4 trimethylation, maintained by CXXC1, and the Lid/KDM5–Sin3A–HDAC1 demethylase complex are required for this transcriptional programme (48, 49). Among ATP-dependent remodellers, CHD4 (a NuRD-complex subunit) represses pro-apoptotic genes in perinatal mouse oocytes; its conditional deletion causes acute loss of non-growing oocytes and ovarian-reserve depletion, giving direct causal evidence that a single chromatin remodeller can gate primordial-follicle survival (50). BPTF (NURF complex) supports porcine granulosa-cell viability via H3K4 methylation (51), and histone methyltransferases SETD1B/SETDB1/SETD2/CFP1 decline with maternal age in mouse oocytes and granulosa cells in parallel with follicle numbers (52).

#### 2.4.2 DNA methylation and histone modifications in ageing ovaries

In aging rat ovary, hypermethylation of the autophagy gene Atg5 is associated with reduced autophagy-related gene and protein expression and inferred activity, rather than a direct measurement of autophagic flux (53), and mouse ovaries show declining global methylation with altered DNMT1/3a/3b expression (54). In aged mouse granulosa cells, increased DNMT1 acetylation reduces DNMT1 protein and causes global hypomethylation and cell-cycle arrest (55). Blood- or follicular-fluid-based DNA-methylation “epigenetic age” correlates with diminished ovarian response and lower oocyte yield in several IVF cohorts (56–58), and is one of the more promising emerging biomarkers discussed further below. The repressive mark H3K27me3 is enriched at variable, differentially methylated CpG sites in mural granulosa cells from women with DOR, including in genes involved in folliculogenesis such as IGF2 and AMH, directly implicating this mark in human DOR (59), and broader histone post-translational modifications, including trimethylation marks, change with age in mouse ovary (60). JMJD3/KDM6b, the demethylase that removes H3K27me3 and regulates mitochondrial genes in granulosa cells, declines with age in both mice and humans (61). FTO-dependent m6A regulation affects FOS mRNA stability in aged human and experimental granulosa-cell settings (62), whereas melatonin-associated YTHDF2 regulation of UBE3C influences p53-linked senescence in mouse and KGN-cell models (63). In human POI, cumulus cells show global hypermethylation/hydroxymethylation that negatively correlates with ovarian-function gene expression (64), and ovarian stimulation/IVM procedures themselves are associated with altered DNA-methylation and chromatin-remodelling gene expression in oocytes, an important confound for any biomarker study performed on stimulated cycles (65, 66).

#### 2.4.3 Non-coding RNAs

miR-144-3p protects granulosa cells from chemotherapy-induced apoptosis and premature primordial-follicle activation via MAP3K9/p38-MAPK/PI3K-AKT (67). LncRNA NEAT1 promotes granulosa-cell proliferation and oestradiol synthesis via miR-204-5p/ESR1/MAPK, with its down-regulation linked to impaired steroidogenesis and apoptosis in human DOR granulosa cells (68). LncRNA GCAT1 is down-regulated in biochemical POI and regulates the cell-cycle inhibitor p27 via competitive binding to PTBP1 (69); lnc-GULP1-2:1 and PWRN1 similarly regulate granulosa-cell proliferation, apoptosis and steroidogenesis (70, 71). Follicular-fluid extracellular-vesicle and miRNA profiles are being explored as non-invasive biomarkers of ovarian ageing and impaired reserve. However, the cited evidence comprises two reviews and one small comparative DOR/PCOS study, so independent diagnostic validation is lacking (72–74).

Several of these findings — NEAT1, GCAT1, lnc-GULP1-2:1, PWRN1, exploratory follicular-fluid extracellular-vesicle profiles, and DNA-methylation epigenetic-age acceleration — have been examined directly in human DOR or POI granulosa cells and follicular fluid. The extracellular-vesicle evidence remains small and unvalidated, and epigenetic-age acceleration has not yet been shown to be a causal driver of reserve depletion rather than a correlate of the same underlying ageing process captured by AMH and AFC. Most of the chromatin-remodeller mechanisms discussed above (CHD4, BPTF, SETD1B/2/DB1, EZH2/JMJD3, FTO/YTHDF2), by contrast, are established in mouse genetic models or porcine and human cell lines and extrapolated to human DOR; disentangling stimulation-induced epigenetic changes from those that pre-date ovarian stimulation remains a broader methodological problem for this literature.

### 2.5 Mitochondrial bioenergetics and oxidative stress

#### 2.5.1 Mitochondrial bioenergetics in oocyte competence

Mitochondria are the oocyte’s principal ATP source via oxidative phosphorylation (OXPHOS), and impaired ATP production, membrane-potential loss and mitochondrial mispositioning (mediated by MIRO1 and the mitofusins MFN1/MFN2) are consistently associated with reduced oocyte developmental competence across species (75–78). Oocyte-specific Mfn1/Mfn2 deletion causes infertility and follicular depletion in mice, directly linking mitochondrial dynamics to reserve (78). NAD+-salvage-pathway support (NAMPT activators and nicotinamide mononucleotide) improves spindle assembly and mitochondrial performance in aged mouse oocytes (79, 80), whereas mitochondrial Ca2+ overload delays meiotic progression (81). Experimental oocyte models also identify PKD, OPA1 and PARL as regulators of mitochondrial turnover and mitophagy (82–84); their quality-control functions are considered in Section 2.6.3. In human DOR granulosa cells, glycolytic enzymes (hexokinase, PGK and LDH1), citrate-synthase activity and glucose transporters GLUT1/3 are reduced together with AMPK signalling, providing direct evidence of a granulosa-cell energy deficit (85).

#### 2.5.2 mtDNA integrity and mitochondrial dynamics

mtDNA damage and altered copy number are frequently invoked as DOR mechanisms, but the human literature is mixed rather than uniformly showing a decrease. An early study of human MII oocytes reported maternal-age-associated mtDNA point mutations, not a copy-number measurement (86). In a Sod2 conditional-knockout model, experimentally elevated mitochondrial ROS impaired oocyte quality and reproductive lifespan primarily by destabilising mtDNA copy number rather than by simply increasing mutation load, and restoration of mtDNA copy number rescued the phenotype (87). A recent review likewise concludes that mtDNA mutation burden and copy-number dysregulation may be more informative than an assumed monotonic decline in copy number (88). Age-related mtDNA deletions in skeletal muscle and fission/fusion control of mtDNA integrity in yeast are retained here only as explicitly labelled cross-tissue or cross-species analogies, not as ovarian evidence (89–91). Ovarian studies of mitophagy and mitochondrial turnover provide additional experimental context (92–95). Human DOR studies identify miR-361-5p/SLC25A24- and HOXA1-associated mitochondrial dysfunction (96, 97), whereas evidence involving SIRT3, CLPP and mitochondrial-DAMP/NLRP3 signalling comes from aging or chemotherapy mouse models, human-cell/POI work and rat models (98–101). Together, these findings establish mitochondrial dysfunction as an important upstream stress axis while linking mitochondrial cargo disposal to the autophagy–lysosome system discussed in Section 2.6.

#### 2.5.3 Oxidative stress and antioxidant defence

Excess ROS arising from OXPHOS, environmental insults, chemotherapy and ageing can damage mtDNA, lipids and proteins and thereby create an upstream stress state that feeds into the apoptosis, ferroptosis and inflammatory programmes discussed in Sections 2.7 and 2.8 (102–106). To avoid conflating oxidative stress with its downstream execution pathways, this subsection focuses on redox defence itself. The mammalian ovarian/granulosa-cell antioxidant system comprises superoxide dismutases (SOD1/2/3), catalase (CAT), glutathione peroxidases (including GPX1/GPX4), peroxiredoxins (PRDX), reduced glutathione (GSH) and the transcription factor Nrf2 (NFE2L2). In human granulosa cells, oxidative stress-dependent release of Nrf2 from KEAP1 up-regulates antioxidant and glutathione-synthesis genes (107). The SIRT1-Nrf2 axis further links antioxidant defence to maintenance of granulosa-cell viability, with GPX4 providing a mechanistic bridge to ferroptosis without making ferroptosis itself part of the oxidative-stress subsection (108). Among non-enzymatic antioxidants, coenzyme Q10 and melatonin have been reviewed most directly in ovarian and assisted-reproduction contexts (109, 110). Hypoxia-conditioned human umbilical-cord mesenchymal-stromal-cell exosomes reduced mitochondrial oxidative stress through SIRT3/PGC1α in cyclophosphamide-treated KGN cells and POI rats (111), whereas 3D-cultured human umbilical-cord mesenchymal-stromal-cell spheroids activated Nrf2 and attenuated ferroptotic markers in granulosa-cell and rat models (112). Ovarian toxicology studies, including benzo[a]pyrene exposure, provide independent evidence that exogenous oxidative insults can compromise ovarian function (113).

Taken together, mitochondrial bioenergetic failure, mtDNA instability and redox imbalance form an interconnected upstream stress axis in DOR. The strongest direct human-DOR evidence in this domain lies in granulosa-cell metabolic dysfunction and selected mitochondrial signatures, whereas many causal mechanisms remain supported primarily by mammalian models. The direction of mtDNA copy-number change in human DOR remains unresolved, and oxidative stress should be distinguished from the downstream apoptosis, ferroptosis and inflammatory programmes that it can trigger. Selective disposal of damaged mitochondria is therefore discussed separately as mitophagy within the autophagy–lysosome system (Section 2.6.3).

### 2.6 Autophagy–lysosome system

The autophagy–lysosome system is a core homeostatic network that couples cytoplasmic cargo sequestration to lysosomal degradation and recycling. In ovarian cells, autophagy is best understood as a quality-control and metabolic-adaptation programme rather than an intrinsically lethal pathway. Its biological effect depends on cell type, follicular stage, stress intensity, cargo identity and, critically, whether autophagic flux is successfully completed. Accordingly, changes in static markers such as LC3 or SQSTM1/p62 should not be interpreted as evidence of increased or decreased flux without complementary functional measurements. For mechanistic clarity, this section distinguishes overall autophagic flux, non-selective bulk autophagy, selective cargo autophagy and lysosomal proteolytic signalling.

#### 2.6.1 Autophagic flux and ovarian homeostasis

Efficient autophagic flux requires coordinated autophagosome formation, maturation, fusion with lysosomes and cargo degradation. Disruption of EPG5, a factor required for late-stage autophagosome–lysosome processing, causes primary ovarian insufficiency in mice through granulosa-cell dysfunction and WT1 accumulation (114). Complementary human and experimental evidence links macroautophagy to granulosa-cell differentiation: genetic suppression of ATG5 or BECN1, or pharmacological inhibition with chloroquine, reduces FSHR and CYP19A1 expression and estradiol synthesis, while impaired autophagy permits WT1 accumulation; granulosa cells from women with biochemical POI also show reduced autophagy-related signatures (115). These findings support autophagic competence as a component of ovarian homeostasis, while also illustrating that direct evidence in age-matched women with DOR remains limited.

#### 2.6.2 Non-selective autophagy

Non-selective, or bulk, macroautophagy sequesters portions of cytoplasm without a defined cargo-specific receptor and is particularly important during nutrient limitation and generalised cellular stress. In ovarian dysfunction models, modulation of this bulk autophagic response can be protective or detrimental depending on context. HO-1-expressing umbilical-cord mesenchymal stromal cells restore ovarian function in a POI mouse model in association with JNK/Bcl-2-regulated autophagy (116), whereas melatonin limits excessive autophagy-associated mitochondrial and ovarian-reserve dysfunction in Chinese hamster ovary cells through ERK signalling (117). Non-coding RNAs add another regulatory layer: NEAT1 and SNHG15 alter granulosa-cell autophagy through distinct MAPK- and FOXO3-related axes in ovarian dysfunction models (118, 119). Because these studies generally manipulate overall autophagic activity without identifying a receptor-defined cargo, they are better interpreted as evidence for context-dependent bulk autophagy than as distinct cell-death mechanisms.

#### 2.6.3 Selective autophagy

Selective autophagy removes defined substrates through cargo-recognition mechanisms and therefore provides a more mechanistically precise link between cellular stress and ovarian quality control. Mitophagy is the best-characterised selective pathway in ovarian biology: Nur77-, PINK1-related and polyamine-sensitive mechanisms support removal of dysfunctional mitochondria during reproductive ageing and oocyte maturation (92–95). Pink1 deletion in mice impairs mitophagy, accelerates follicle depletion and ovarian ageing, and reduces oocyte quality, with substantially smaller ovarian effects after Parkin deletion, suggesting that ovarian PINK1-dependent quality control is not obligatorily Parkin-dependent (120). A 2026 study of granulosa cells from normoresponder and DOR IVF cohorts identified group- and oocyte-maturation-associated differences in selected mitochondrial quality-control markers, providing direct but observational human evidence rather than proof of mitophagic flux or causality (121).

Stress-granule autophagy (granulophagy) has recently emerged as another ovarian stress-resilience mechanism. NCOA7 functions as a selective autophagy adaptor that promotes G3BP1–V-ATPase-dependent clearance of persistent stress granules; reduced NCOA7 is associated with granulosa-cell senescence in pathological and physiological ovarian ageing, whereas enhancing granulophagy alleviates senescence in human granulosa cells and delays ovarian ageing in mice (122). Lipophagy provides a third ovary-relevant selective route: in human ovarian tissue and granulosa-cell models, autophagic delivery of lipid droplets to lysosomes releases cholesterol for steroidogenesis, and disruption of this pathway reduces sex-steroid production (123). Other selective pathways, including ER-phagy, aggrephagy and pexophagy, are established cellular quality-control programmes but currently lack sufficiently direct ovarian-reserve evidence to be considered established DOR mechanisms.

#### 2.6.4 Lysosomal function and proteolytic signalling

Lysosomal competence is the terminal determinant of whether autophagic cargo is actually degraded rather than merely sequestered. The EPG5 phenotype above highlights the importance of autophagosome–lysosome processing (114), while the NCOA7 mechanism links V-ATPase-dependent lysosomal function to stress-granule clearance (122). Cathepsin B further connects lysosomal proteolysis to ovarian-reserve regulation by controlling IGF1R turnover and downstream AKT–mTOR signalling; its inhibition increases IGF1R abundance, alters mitochondrial biogenesis and mitophagy programmes, and preserves ovarian-reserve quantity and quality in preclinical models (124). This evidence supports a lysosomal proteostasis/signalling role rather than proof of lysosomal membrane-permeabilisation-dependent cell death in human DOR. Overall, the autophagy–lysosome system should be viewed as a bidirectional gatekeeper of ovarian proteostasis: insufficient cargo clearance, defective lysosomal processing and pathological overactivation may each be detrimental, but physiological autophagy principally supports survival and adaptation. The integrated relationships among mitochondrial bioenergetics, autophagy-lysosome system and oxidative stress are summarised in **Figure 3**.

**Figure 3:**
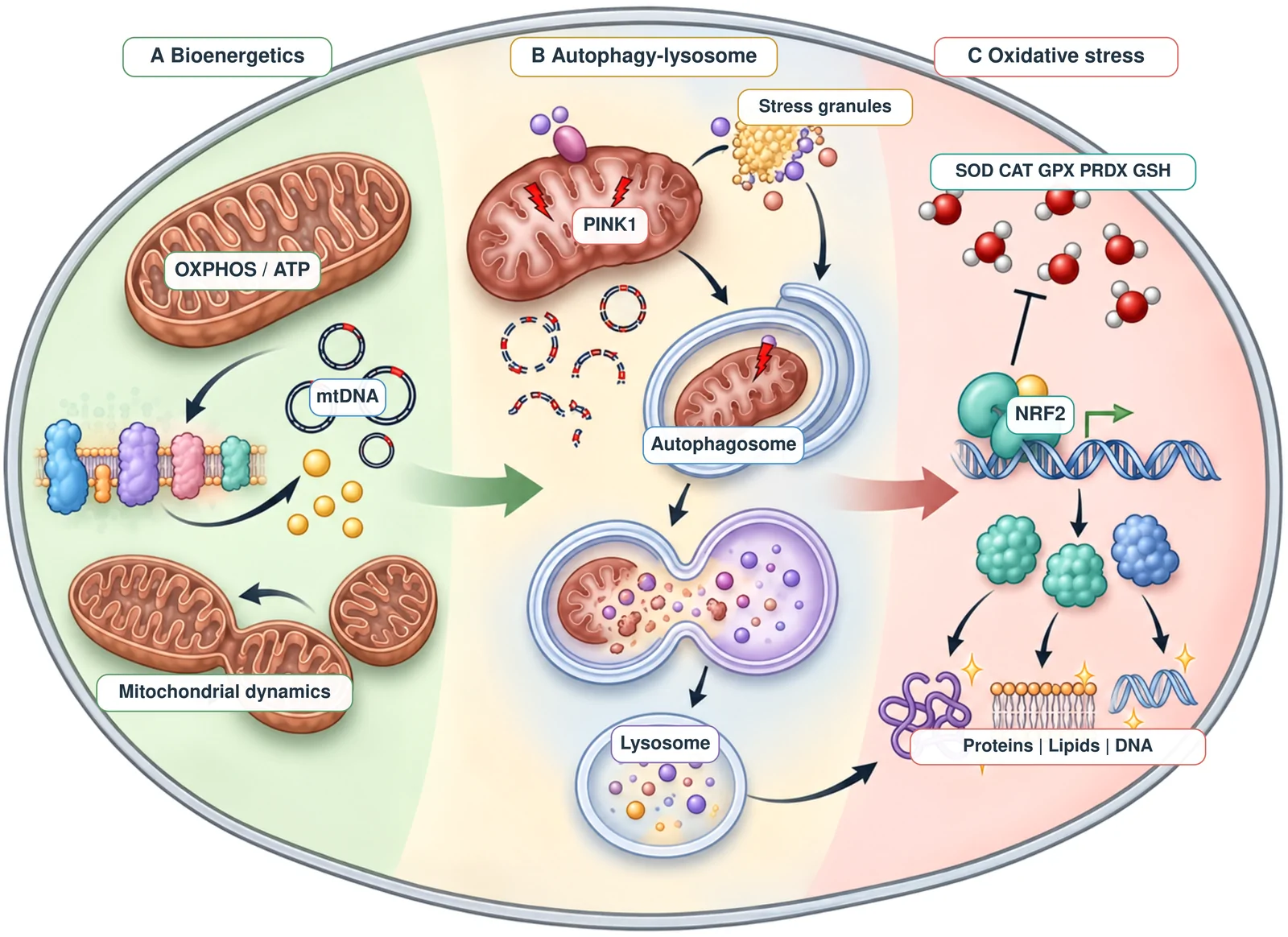
Mitochondrial bioenergetics, autophagy-lysosome system and oxidative stress. **(A)** Mitochondrial oxidative phosphorylation, mtDNA integrity and mitochondrial dynamics support ATP production and oocyte competence. **(B)** The autophagy-lysosome system removes damaged cellular components through pathways including PINK1-associated mitophagy and stress-granule clearance, followed by autophagosome-lysosome degradation. **(C)** Excess reactive oxygen species (ROS) are counteracted by antioxidant systems including NRF2, SOD, CAT, GPX, PRDX and GSH; failure of this defence promotes oxidative damage to proteins, lipids and DNA. Together, mitochondrial dysfunction, impaired cellular quality control and redox imbalance contribute to ovarian dysfunction.

### 2.7 Cell fate, senescence and regulated cell death

This section separates bona fide regulated cell-death programmes from non-lethal cell-fate states. Apoptosis and ferroptosis have the strongest mechanistic support in ovarian follicle loss; cellular senescence is included because it durably alters granulosa-cell fate and the follicular microenvironment but is not itself a form of cell death. Other recently described death modalities are presented as emerging or hypothesis-generating mechanisms when direct human DOR evidence is lacking. Autophagy is addressed separately in Section 2.6 as a context-dependent quality-control pathway rather than an intrinsically lethal process.

#### 2.7.1 Apoptosis

Intrinsic mitochondrial apoptosis (increased Bax/cleaved caspase-3 and decreased Bcl-2) is supported by mouse DOR experiments and oxidant-treated COV434 human granulosa-tumour cells (125, 126); these two studies are not direct human DOR evidence. PI3K/AKT dysregulation can remove pro-survival signalling in DOR (127). In human DOR granulosa cells, miR-484/YAP1 and miR-221-3p/FOXO1 have been linked to apoptotic regulation (128, 129). Chemotherapy-induced reserve loss can involve follicular overactivation, stromal injury and direct follicular toxicity, including apoptotic pathways, but the cited source is a mechanistic review rather than primary DOR data (130). An aging-mouse GH study implicates Fos/Jun signalling but is neither chemotherapy evidence nor a human DOR study and is considered in Section 3.2 (131). Additional TGF-beta/SMAD, Hippo-YAP and Wnt crosstalk — including miR-361-5p/VEGFA, broader granulosa-cell-death networks, miR-6881-3p/SMAD4 in human DOR, miR-34a-5p/LEF1, SMAD4/FZD4 and Gremlin-1-dependent signalling — further modulates granulosa-cell apoptosis across ovarian models (132–137). Oxidative stress is an important upstream trigger, but its redox biology is not repeated here.

#### 2.7.2 Cellular senescence

Distinct from apoptotic cell death, granulosa cells and oocytes can enter a senescent, non-dividing but metabolically active state marked by p16INK4a/p21CIP1 up-regulation and a pro-inflammatory secretory phenotype. Hallmarks of cellular ageing emerge in the mouse ovary before overt primordial-follicle depletion, suggesting that senescence may precede, rather than merely follow, follicle loss (138). Targeting cellular senescence is therefore an active area of ovarian-ageing research (139). Senescence also links the major mechanisms reviewed above: DNMT1 acetylation-driven hypomethylation connects it to epigenetic ageing (55), whereas USP14 inhibition links reduced senescence to improved DNA-damage repair in a POI model (140). The NCOA7 stress-granule-clearance mechanism described in Section 2.6.3 adds a newer proteostasis-based route through which chronic cellular stress may promote granulosa-cell senescence.

#### 2.7.3 Ferroptosis

Ferroptosis is an iron-dependent, lipid-peroxidation-driven regulated cell-death programme that depends on failure of the cystine-glutathione-GPX4 defence system (141). SLC7A11 is the light-chain subunit of the cystine/glutamate antiporter system Xc- and should not be described as an iron or copper importer; it exchanges extracellular cystine for intracellular glutamate, supplying cysteine for glutathione synthesis and thereby supporting GPX4-mediated detoxification of lipid peroxides (142). In human DOR granulosa cells, GPX4 expression and glutathione levels are reduced, with accompanying mitochondrial abnormalities consistent with ferroptotic stress (143). The ATF4/SLC7A11/GPX4 axis is supported in a premature-ovarian-failure model (144), while the SIRT1-Nrf2 pathway discussed in Section 2.5.3 converges on GPX4 (108). Keeping the redox-defence discussion upstream and the lethal lipid-peroxidation programme here reduces overlap between oxidative stress and ferroptosis while preserving their mechanistic connection.

#### 2.7.4 Emerging regulated cell-death modalities

Several additional regulated cell-death modalities have been proposed in ovarian dysfunction but currently lack direct human DOR evidence and should remain hypothesis-generating. Cuproptosis is driven by copper accumulation, FDX1-dependent mitochondrial copper handling and aggregation of lipoylated TCA-cycle proteins; copper uptake is mediated by SLC31A1/CTR1 rather than SLC7A11 (145, 146). A decabromodiphenyl-ether mouse model provides ovarian evidence for activation of this pathway and melatonin-mediated protection (147), but this remains preclinical toxicant-model evidence. Necroptosis (RIPK1/RIPK3/MLKL) has been associated with reduced follicle counts after air-pollution exposure in rodents (148). Oxeiptosis (KEAP1/PGAM5/AIFM1) has been modulated in a single cyclophosphamide-induced rat DOR study (149), and PANoptosis involving a ZBP1/RIPK3/RIPK1 complex has been described in porcine granulosa cells (150). Pyroptosis is considered primarily in the immune-inflammatory section because its ovarian evidence is closely tied to inflammasome signalling. Lysosomal-autophagic regulation through Cathepsin B is addressed in Section 2.6.4 and is not classified here as lysosomal cell death, because the available ovarian study supports reserve regulation through lysosomal proteolysis, IGF1R turnover and mitophagy rather than demonstrating lysosomal membrane-permeabilisation-dependent death. The evidence-stratified cell-fate pathways are summarised in **Figure 4**.

**Figure 4:**
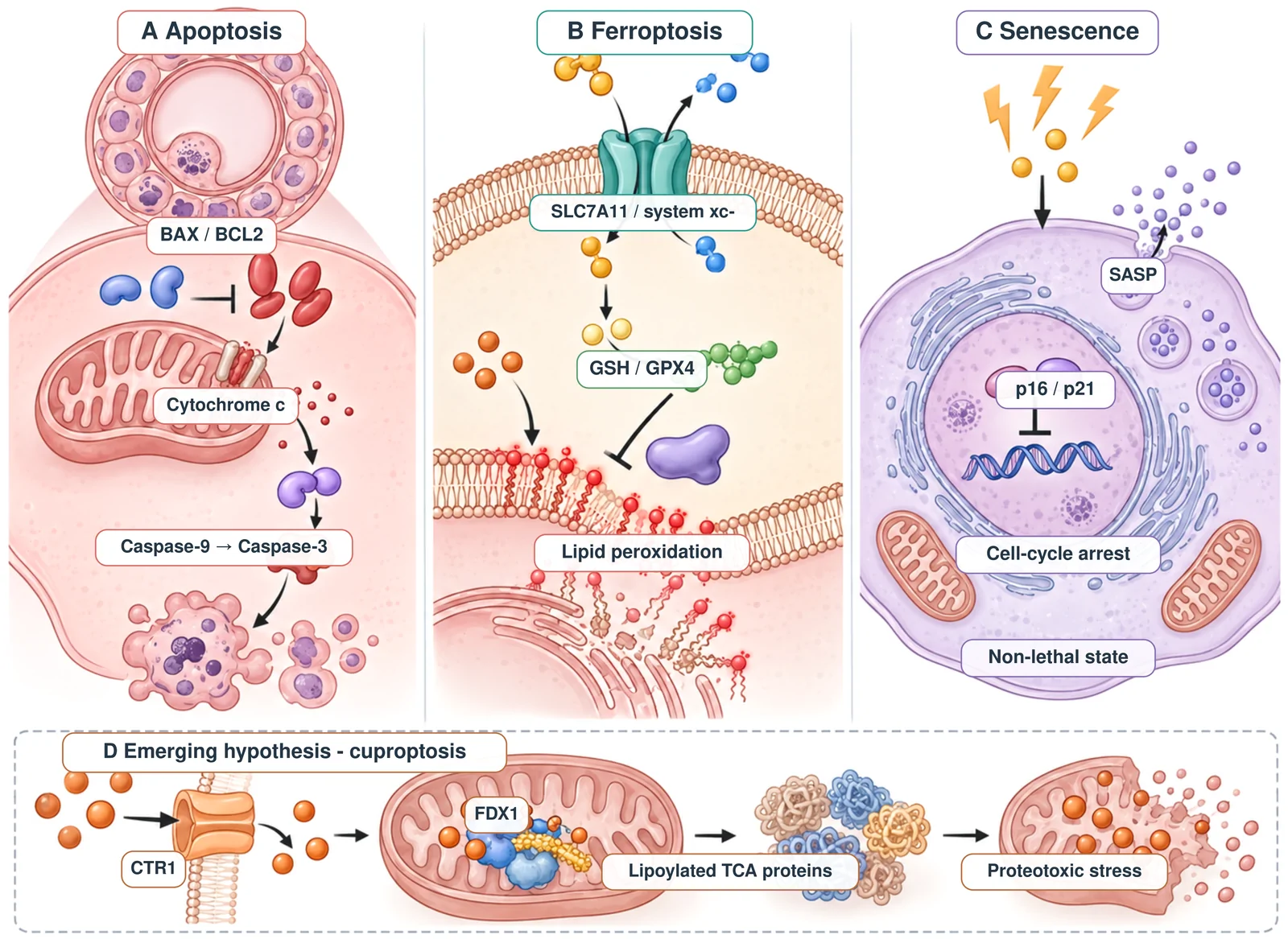
Cell fate, senescence and regulated cell death in ovarian dysfunction. **(A)** Intrinsic apoptosis involves BAX/BCL2 imbalance, mitochondrial cytochrome-c release and caspase activation. **(B)** Ferroptosis results from failure of the SLC7A11-GSH-GPX4 antioxidant axis and consequent lipid peroxidation. **(C)** Cellular senescence is a non-lethal state characterised by p16/p21-associated cell-cycle arrest and a senescence-associated secretory phenotype (SASP). **(D)** Cuproptosis is presented as an emerging mechanism involving CTR1-mediated copper uptake, FDX1-associated mitochondrial metabolism and aggregation of lipoylated TCA-cycle proteins. Apoptosis and ferroptosis have stronger ovarian evidence, whereas the contribution of emerging regulated cell-death pathways to human DOR requires further validation.

### 2.8 Immune and inflammatory signalling

Autoimmune POI represents a mechanistically and clinically distinct entity from general age-related DOR, and several proposed regenerative mechanisms — ovarian germline stem cells and gut-microbiota manipulation — remain considerably more controversial than their frequent citation in the translational literature would suggest; both distinctions are maintained explicitly throughout this section.

#### 2.8.1 The ovarian immune niche

Macrophages, T cells, NK cells and B cells and their cytokines participate in normal follicle turnover, and their dysfunction is implicated in reserve decline. Aged-mouse ovarian macrophages show reduced phagocytic clearance of apoptotic debris, fostering local inflammation (151). Multiparity in aged mice promotes primordial-follicle retention via enhanced local/systemic innate immunity (CXCL1/2, CSF3) (152), while deficiency of vasoactive intestinal peptide (VIP) produces fibro-inflammatory ovarian changes and foamy-macrophage accumulation (153). Genetic macrophage ablation rescues ovarian function in a zebrafish model of macrophage-driven ovarian failure, establishing a germline–somatic–macrophage axis as a plausible, though non-mammalian, mechanism (154). In a nine-woman single-cell study, gamma-delta T cells were reduced and VEGFA-FLT1 interaction between monocytes and granulosa cells appeared lost in POI and advanced maternal age compared with controls (155).

#### 2.8.2 Chronic low-grade inflammation

Human POI cohorts show increased follicular-fluid IL-33 and reduced soluble ST2 (156), and human DOR data support ROS-CCL5-mediated CD8+ T-cell recruitment and granulosa-cell apoptosis (157). Separately, Nlrp3- and Asc-knockout experiments in mice implicate inflammasome-dependent inflammation in age-related ovarian-reserve depletion (158). Genetic variants in the pro-inflammatory receptor GPR84 have been linked to non-age-related DOR, suggesting a heritable component to inflammation-driven reserve decline (159). NLRP3-inflammasome-mediated pyroptosis is one specific mechanism by which the endocrine-disrupting phthalate DEHP damages granulosa cells via the SLC39A5/NF-κB/NLRP3 axis in mice (160). Vitamin D has well-established general immunoregulatory functions, but direct evidence linking vitamin D status to human ovarian reserve remains limited, and general immunology data on vitamin D are not extended here to an ovarian-specific conclusion.

#### 2.8.3 Autoimmune oophoritis

Autoimmune POI is a recognised subtype in which lymphocytic oophoritis and autoantibodies against steroidogenic enzymes can occur. Steroidogenic-cell or adrenal-cortex autoantibodies identify a minority of cases; one prospective cohort found adrenal-cortex autoantibodies in 10 of 266 women (3.8%) (161). A T-helper-1/regulatory-T-cell imbalance mediates granulosa-cell apoptosis via IFN-γ/TNF-α in this specific subgroup, and IDO-mediated restoration of Treg proportions improves ovarian function in a POI model (162, 163). Reviews note that broader estimates vary by subtype, case definition and assay; this autoimmune mechanism should not be generalised to age-related DOR, in which autoantibody positivity is not a typical finding (164).

#### 2.8.4 Ovarian germline stem cells and gut microbiota

Claims that adult mammalian ovaries retain germline stem cells (GSCs/OSCs) capable of neo-oogenesis remain highly controversial. The original report of mitotically active, DDX4-positive putative oogonial stem cells in reproductive-age women (165) prompted both supportive and strongly rebutting follow-up work; a 2023 study explicitly titled “The Continued Absence of Functional Germline Stem Cells in Adult Ovaries” reports failure to find functional GSC activity, and reviews of the field describe the underlying isolation methodology and marker specificity (DDX4) as still contested (166, 167). Chito-oligosaccharide/macrophage-mediated “ovarian stem cell” proliferation findings (168) should accordingly be regarded as preliminary and disputed rather than as an established regenerative mechanism. Similarly, microbiome links to POI are primarily observational in human reproductive-tract studies or synthesised from preclinical gut-microbiota experiments; the cited reviews do not establish controlled human efficacy of faecal microbiota transplantation or probiotic supplementation for DOR/POI (169, 170).

Overall, direct human evidence in this section includes follicular-fluid IL-33/sST2 changes in POI, ROS-CCL5-mediated CD8+ recruitment in DOR, GPR84 variants, and steroidogenic/adrenal autoantibodies in the autoimmune-POI subtype. By contrast, macrophage-clearance and VIP-deficiency data derive from aged mice, and the proposed germline–somatic–macrophage axis rests on a zebrafish model. The existence and functional relevance of adult ovarian germline stem cells remains scientifically contested, and microbiota-directed treatment of ovarian reserve has not been clinically validated by the cited evidence.

### 2.9 Endocrine and systemic modulation

DOR is also shaped by signals acting on the ovary from outside it. The secreted protein C1QL1 regulates follicle depletion through combined intraovarian and endocrine (hypothalamic KISS1/GnRH, pituitary FSH) control; C1ql1-deficient mice show excessive primordial-follicle recruitment and accelerated depletion (171). Iron-overload-driven hypogonadotropic hypogonadism in transfusion-dependent β-thalassaemia major is a genuinely human, if population-specific, example of systemic disease impairing ovarian reserve via hypothalamic-pituitary rather than intraovarian mechanisms (172). Metabolic disease (insulin resistance, obesity), particularly when co-occurring with PCOS, is associated with altered reserve markers in Chinese DOR-with-PCOS cohorts (173). Chronic stress activates the hypothalamic-pituitary-adrenal axis and the ovarian renin-angiotensin system (ACE-AngII-AT1R), increasing granulosa-cell oxidative stress and apoptosis; the antioxidant chlorogenic acid ameliorates this in a chronic-unpredictable-stress rodent model (174).

## 3 Clinical translation and therapeutic perspectives

This section connects the mechanistic material above to biomarkers and interventions used, or proposed for use, in clinical practice (**Table 2**).

**Table 2:** Current clinical evidence for candidate biomarkers and therapeutic interventions in diminished ovarian reserve.

Biomarker/intervention	Best-supported effect	Live-birth evidence in DOR	Current clinical status
AMH/AFC	Predicts oocyte yield after stimulation (ref. 1)	Poor independent predictor of pregnancy chance (guideline-stated)	Established diagnostic marker; not a fertility pass/fail test
DNA-methylation epigenetic age	Correlates with oocyte yield in IVF cohorts (refs. 58–60)	Not tested	Investigational; no prospective live-birth validation
Follicular-fluid extracellular-vesicle/miRNA profiles	Exploratory profiles; one small comparative primary study (refs. 74–76)	Not tested	Early-stage; no independent diagnostic validation
Dehydroepiandrosterone (DHEA)	Oocyte number; inconsistent across meta-analyses (refs. 178–180)	Not established	Investigational adjuvant; conflicting trial evidence
Testosterone pretreatment	Oocyte number (ref. 180)	Higher live-birth rate in one 2025 RCT meta-analysis	Investigational adjuvant
Growth hormone adjuvant	Positive 2020 POR meta-analysis; no significant benefit in the 2025 POSEIDON synthesis (refs. 180, 181)	Mixed meta-analytic evidence	Investigational; not established standard care
Coenzyme Q10/melatonin	Oxidative-stress and mitochondrial endpoints (refs. 111, 112)	Not established	Investigational; largely surrogate-endpoint evidence
*In vitro* activation (Hippo/AKT disruption + fragmentation)	Follicle growth and oocyte retrieval (ref. 12)	Isolated live births reported in POI, not DOR, patients	Investigational; evidence base is a small POI series
Mesenchymal-stromal-cell/extracellular-vesicle approaches	Exosomes: SIRT3/PGC1α; 3D spheroids: Nrf2/ferroptosis (refs. 113, 114)	None	Preclinical
Putative ovarian germline stem-cell activation	Disputed (refs. 167–170)	None	Preclinical and scientifically contested
Gene editing	No DOR-directed ovarian efficacy study among the cited literature	None	Distant research direction, not a near-term option
Gut-microbiota transplantation/probiotics	Observational human links and preclinical intervention evidence (refs. 171, 172)	None	Not clinically validated for DOR/POI
Fertility preservation (oocyte/embryo cryopreservation)	Banks oocytes or embryos before further reserve decline (ref. 4)	Established indirect benefit	Established clinical practice

### 3.1 Biomarkers of ovarian reserve

AMH and AFC remain the clinically established, guideline-endorsed markers of ovarian reserve; per the ASRM committee opinion, both predict oocyte yield well but predict spontaneous pregnancy chance poorly and must not be used to counsel a normally cycling woman that she cannot conceive (1). Cholesterol-metabolism markers are reduced in DOR granulosa cells and follicular fluid and have been proposed as adjunct biomarkers; this remains a single-cohort observation without prospective validation (175). DNA-methylation-based “epigenetic age” is the most promising emerging biomarker class, with several independent IVF cohorts showing correlation with oocyte yield (56–58), but it has not been prospectively validated against live birth and is not presently more informative than AMH/AFC for individual patient counselling. Follicular-fluid extracellular-vesicle and miRNA profiles remain exploratory; the cited primary dataset is small and lacks independent validation (72–74).

### 3.2 Therapeutic interventions

Interventions discussed below may improve selected ovarian-response or cellular endpoints (oocyte number, AMH change, biochemical markers) in some studies; none has been shown in adequately powered randomised trials to increase live birth specifically in women with DOR, and this is, in our assessment, the single most important clinical-translation conclusion of this review.

Dehydroepiandrosterone (DHEA) is the most extensively trialled adjuvant. A 2013 meta-analysis of the (at the time, few) controlled DOR trials found no significant benefit for clinical pregnancy or miscarriage and a significantly lower number of oocytes retrieved with DHEA (176). A later meta-analysis reported significant increases in oocytes retrieved and clinical pregnancy rate (177), and a 2025 systematic review/meta-analysis of RCTs in POSEIDON-defined DOR concluded that DHEA, testosterone, high-dose gonadotropin and a delayed-start antagonist protocol significantly improved oocyte yield; testosterone additionally showed higher live-birth and clinical-pregnancy rates than no supplementation (178). Taken together, the evidence for DHEA is inconsistent across meta-analyses and, where positive, supports an effect on oocyte yield rather than a demonstrated live-birth benefit specific to DOR.

Coenzyme Q10, melatonin and other antioxidants have consistent mechanistic rationale and improve oocyte mitochondrial/oxidative-stress markers in small trials and animal models (109, 110), but — as for DHEA — controlled evidence for a live-birth benefit specific to DOR is not established.

The clinical evidence for growth hormone (GH) is mixed across meta-analyses. A 2020 meta-analysis of 15 randomised controlled trials in poor ovarian responders (1,448 patients) reported improved live-birth rate, clinical-pregnancy rate and oocyte number and fewer cancelled cycles (179). By contrast, the 2025 POSEIDON-restricted synthesis found significant gains in oocyte yield for testosterone, DHEA and delayed-start protocols, and fewer oocytes with low- than high-dose gonadotropin, but no significant GH benefit; only testosterone showed a significant live-birth advantage (178). A mouse study suggests a potential mechanism through suppression of Fos/Jun signalling in ageing ovaries (131), but it does not resolve clinical efficacy. GH therefore remains an investigational adjuvant rather than an established standard of care, and larger phenotype-specific trials are needed.

*In vitro* activation (IVA) — Hippo-pathway disruption plus AKT stimulation — has produced isolated live births in POI patients undergoing autotransplantation (10), but this is case-series-level evidence in a POI, not a DOR, population, and has not been tested against control in an adequately powered DOR trial.

Gene editing and cell-based or extracellular-vesicle approaches — including hypoxia-conditioned human umbilical-cord mesenchymal-stromal-cell exosomes targeting SIRT3/PGC1α (111), 3D mesenchymal-stromal-cell spheroids affecting Nrf2/ferroptosis (112), and putative ovarian germline stem cells (165–168) — remain preclinical or scientifically contested. We explicitly position gene editing as a distant research direction rather than a near-term clinical option: no DOR-directed ovarian gene-editing intervention is represented in the cited clinical literature, and preclinical work has not yet resolved the off-target, mosaicism and germline-heritability concerns required for translation. Immunomodulatory approaches (Treg restoration, IDO induction) are similarly preclinical and specific to the autoimmune-POI subtype, not general DOR.

### 3.3 Fertility preservation and clinical feasibility

For women identified with DOR, the two interventions with the clearest evidence base remain conventional: earlier referral for fertility treatment given the time-sensitive nature of reserve decline, and individualised ovarian-stimulation protocols informed by POSEIDON stratification (3, 180) rather than a one-size-fits-all approach. Fertility-preservation counselling (oocyte/embryo cryopreservation) should be offered proactively where DOR is identified in women not yet ready to conceive, while distinguishing DOR from POI, in which spontaneous pregnancy remains possible (181), since this is the only intervention in this section with a well-established mechanism of benefit (banking oocytes at a higher-reserve time point) rather than an attempt to reverse an already-diminished reserve. The principal feasibility challenges are the cost and access barriers to ART and cryopreservation, the current absence of a validated biomarker that improves on AMH/AFC for individual counselling, and the risk — evident in some of the literature reviewed above — of overselling mechanistic, cellular-endpoint findings as near-term clinical solutions before they have been tested against live birth.

## 4 Conclusion and future perspectives

Ovarian reserve is best understood not as a single depleting number but as the net output of several interacting biological processes converging on two cell types — the oocyte and the granulosa cell (**Figure 1**). Follicle-activation signalling (PI3K-AKT-FOXO3/PTEN-mTOR, Hippo-YAP, KIT/KITL, AMH) sets the rate at which the primordial pool is drawn down; genomic-stability, chromatin/epigenetic, and mitochondrial/oxidative-stress mechanisms determine whether an activated follicle’s oocyte and granulosa cells remain competent; and regulated cell death, immune-inflammatory signalling, and endocrine/systemic inputs determine whether a compromised follicle is eliminated or persists in a degraded state. Oxidative stress and mitochondrial dysfunction are the most-connected nodes in this network, sitting mechanistically upstream of DNA damage, several death pathways, and inflammasome activation, which may explain why antioxidant strategies recur across so much of the intervention literature despite inconsistent live-birth evidence.

Direct evidence in clinically defined DOR is concentrated in a limited set of genetic findings (biallelic BRCA1 variants and variants in HFM1 and KASH5; 38, 41, 42), granulosa-cell apoptosis regulators (miR-484/YAP1, miR-221-3p/FOXO1 and miR-6881-3p/SMAD4; 128, 129, 134), NEAT1 dysregulation (68), ROS-CCL5-mediated CD8+ T-cell recruitment (157), and selected granulosa-cell mitochondrial, redox, metabolic and mitophagy-associated observations (miR-361-5p/SLC25A24, HOXA1, GPX4/GSH, mitophagy-associated expression and cholesterol metabolism; 98, 99, 110, 143, 175). Several of these studies combine patient samples with cell or mouse experiments, so their mechanistic intervention components should not be conflated with human causality. Evidence for BRCA-associated treatment vulnerability, RNF111, GCAT1 and most senescence mechanisms derives from broader ovarian-ageing or POI contexts (37, 41, 69, 122, 138–140) and should not be presented as equivalent to DOR-specific evidence. Primordial-follicle-activation signalling, most mitochondrial-quality-control mechanisms and the newer, less-established cell-death modalities discussed above remain substantially inferred from mouse, porcine, bovine or non-mammalian systems. This asymmetry, rather than any single pathway, represents the field’s central limitation, and future studies should prioritise closing it — for example, by testing whether Hippo/AKT-pathway markers or validated mitophagy-flux measurements differ in human DOR versus age-matched normal-reserve ovarian tissue, rather than continuing to add new candidate pathways from animal models alone.

Several important questions remain unanswered. It is not yet clear whether DOR represents an accelerated version of physiological ovarian ageing or a mechanistically distinct entity, since few studies directly compare age-matched DOR against normal-reserve women rather than DOR against generically “aged” tissue. Which of the mechanisms discussed above is rate-limiting in each woman, and whether this differs by aetiology (idiopathic, genetic, autoimmune, iatrogenic, or environmental), is similarly unresolved. Whether mtDNA copy number rises, falls, or becomes dysregulated in either direction in human DOR oocytes, and how this trajectory compares with physiological ageing, remains an open methodological question, as does whether any of the newer cell-death modalities discussed make an additive contribution to human follicle loss or largely reflect toxicant-model phenomena of limited physiological relevance. Finally, whether any biomarker beyond AMH and AFC can be prospectively shown to improve prediction of live birth, rather than oocyte yield alone, has yet to be demonstrated.

Among the biomarkers and targets discussed in this review, DNA-methylation epigenetic-age scores, evaluated across several IVF cohorts against ovarian-response or oocyte-yield endpoints, are near-term candidates for prospective live-birth validation (56–58). GPX4/ferroptosis-pathway activity and the SIRT1–NRF2 axis, which converge mechanistically with oxidative stress, are comparatively well-supported cellular-endpoint targets, although translation into an intervention that improves live birth has not been demonstrated (108, 143). Targeted genetic evaluation—including established FMR1 testing and context-dependent interpretation of BRCA1/2, HFM1, KASH5 and RNF111 findings—can inform counselling and fertility-preservation discussions in appropriately selected patients (36, 37, 39–45).

Future research would benefit from prospective, age-matched studies comparing DOR against normal reserve for the mechanisms that currently rest on animal models alone, particularly follicle-activation signalling and mitophagy; from adequately powered randomised trials that use live birth, rather than oocyte number or AMH change, as the primary endpoint for DHEA, growth hormone, and antioxidant adjuvants, given the inconsistency documented above; from mechanistic studies that directly test, rather than assume, the contribution of the newer cell-death modalities discussed above to naturally occurring, as opposed to toxicant-induced, follicle loss; from resolution of the ovarian-germline-stem-cell controversy through pre-registered, independently replicated lineage-tracing studies before further therapeutic development in that direction; and from integration of single-cell and spatial-omics data across the stromal, immune, vascular and follicular compartments to test whether stromal ageing is causal or consequential to follicle loss.

Understanding the molecular determinants of ovarian reserve remains an important goal for reproductive medicine. Progress toward that goal will be better served by an evidence base that is explicit about the difference between a mechanism demonstrated in a woman with DOR and one inferred from a mouse, a toxicant model, or another organ system, than by a literature that treats all such evidence as interchangeable.
